# Transcriptional Expression of the *ompA*, *cpaf*, *tarp*, and *tox* Genes of *Chlamydia trachomatis* Clinical Isolates at Different Stages of the Developmental Cycle

**DOI:** 10.3390/microorganisms7060153

**Published:** 2019-05-28

**Authors:** Suvi Korhonen, Kati Hokynar, Laura Mannonen, Jorma Paavonen, Eija Hiltunen-Back, Mirja Puolakkainen

**Affiliations:** 1 Department of Virology, University of Helsinki and Helsinki University Hospital, FI-00014 Helsinki, Finland; kati.hokynar@helsinki.fi (K.H.); laura.mannonen@hus.fi (L.M.); mirja.puolakkainen@helsinki.fi (M.P.); 2 Department of Obstetrics and Gynecology, University of Helsinki and Helsinki University Hospital, FI-00029 HUS Helsinki, Finland; jorma.paavonen@helsinki.fi; 3 Clinic of Venereal Diseases, Skin and Allergy Hospital, Helsinki University Hospital, FI-00029 HUS Helsinki, Finland; eija.hiltunen-back@hus.fi

**Keywords:** *Chlamydia trachomatis*, transcriptional expression, low-passage-number isolates

## Abstract

The transcriptional gene expression patterns of *Chlamydia trachomatis* have mainly been studied using reference strains propagated in cultured cells. Here, using five low-passage-number *C. trachomatis* clinical isolates that originated from asymptomatic or symptomatic female patients, the in vitro expression of the *ompA*, *cpaf*, *tarp*, and *tox* genes was studied with reverse transcriptase real-time PCR during the chlamydial developmental cycle. We observed dissimilarities in the gene expression patterns between the low-passage-number clinical isolates and the reference strains. The expression of *ompA* and the peak of the *tox* expression were observed earlier in the reference strains than in most of the clinical isolates. The expression of *cpaf* was high in the reference strains compared with the clinical isolates at the mid-phase (6–24 hours post infection) of the developmental cycle. All of the strains had a rather similar *tarp* expression profile. Four out of five clinical isolates exhibited slower growth kinetics compared with the reference strains. The use of low-passage-number *C. trachomatis* clinical isolates instead of reference strains in the studies might better reflect the situation in human infection.

## 1. Introduction

*Chlamydia trachomatis* urogenital infection is the most common sexually transmitted bacterial infection, affecting annually over 100 million individuals worldwide [1]. Most infections remain asymptomatic, and untreated or repeated infections can result in pelvic inflammatory disease and severe reproductive complications such as infertility and ectopic pregnancy [2]. Based on the *ompA* gene encoding the major outer membrane protein (MOMP), *C. trachomatis* can be classified in genotypes (A-C, D-K, and L1-L3), of which genotypes D-K cause most of the urogenital infections [3].

*C. trachomatis* is an intracellular pathogen with a special two-phase developmental cycle during which the bacteria occur either as infectious elementary bodies (EBs) or as metabolically active reticulate bodies (RBs) [4]. Temporal expression of *C. trachomatis* genes corresponds to the phases of the chlamydial developmental cycle [5,6,7]. Early genes are expressed within one to three hours after endocytosis, mid-cycle genes start expressing during RB replication (6–24 h post infection (hpi)), and late genes are transcribed when the RBs convert back to EBs (24–48 hpi).

During productive infection, *C. trachomatis* interacts with the host cells through the effector proteins that facilitate the bacterial pathogenicity. Among the most extensively studied chlamydial effector proteins are translocated actin-recruiting phosphoprotein (Tarp, CT456), *Chlamydia* protease-like activity factor (CPAF; CT858) and chlamydial cytotoxin (CT166). Tarp is translocated into the host cell by the chlamydial type III secretion system and tyrosine phosphorylated by host kinases [8]. Tarp is involved in signaling events, leading to actin recruitment and to the internalization of the EB, and promoting the intracellular survival of *Chlamydia* [9]. CPAF is a potent type II secreted protease detected in the host cell cytosol [10,11]. Many of the previously reported effects of CPAF have been questioned [12], but CPAF seems to have a role during the exit of EBs [11]. The chlamydial cytotoxin inactivates Rho GTPase Rac1 by glycosylation, inducing the actin depolymerization of the host cell [13,14]. In addition, cytotoxin is involved in delayed cell cycle progression [15].

Most *C. trachomatis* gene expression studies have been performed using reference strains propagated in the laboratory [6,7]. Long-term laboratory propagation of *C. trachomatis* changes the growth properties and gene expression [16,17,18]. To our knowledge, only the expression of *C. trachomatis ompA* has been analyzed using both clinical and reference strains throughout the developmental cycle [19]. The study concluded that the expression levels of clinical type E strains and reference strain type E did not correlate. Our aim was to study the expression of *C. trachomatis ompA*, *cpaf*, *tarp*, and *tox* genes during in vitro infection in the cervical epithelial cell line (HeLa229), using low-passage-number clinical isolates. In addition, we wanted to evaluate the expression of the same genes in the cervical swab specimens collected from the patients with *C. trachomatis* infection.

## 2. Materials and Methods

### 2.1. Patients and Samples

During 2009–2011, 127 female patients attending the outpatient sexually transmitted infection (STI) clinic of Helsinki University Hospital, Helsinki, Finland, and gave informed consent and were enrolled to this study. The patients visited the clinic because of symptoms, for follow-up, or because of notification by an infected partner. A cervical swab was transported in Aptima Swab Transport Medium for *C. trachomatis* and *Neisseria gonorrhoeae* testing at HUSLAB (Aptima Combo 2 Assay, Hologic, Marlborough, MA, USA) Another cervical swab was placed in Universal Transport Medium (UTM) (Copan) for the *C. trachomatis* culture. Of the 127 specimens, 50 were *C. trachomatis* nucleic acid amplification test (NAAT) positive. The study was conducted in accordance with the Declaration of Helsinki, and the protocol was approved by the Independent Institutional Review Board of the Hospital District of Helsinki and Uusimaa (15.6.2009; HUSLAB §44/2009) and the Ethics Committee of the Department of Medicine, Hospital District of Helsinki and Uusimaa (5.6.2999; §241/2009).

### 2.2. C. trachomatis Culture

The anonymized cervical swabs were cultured in McCoy cells (from Pekka Saikku). The cells in 24-well plates were inoculated with UTM and centrifuged at 3000× *g* at 30 °C for one hour. BHK-21 medium supplemented with 10% fetal calf serum, 2 mM glutamine, 20 μg/mL gentamicin, 50 U/mL nystatin, 100 μg/mL vancomycin, and 0.5 μg/mL cycloheximide was added, and the plates were incubated in 5% CO_2_ at 35 °C for 48 hours. The cells were collected in sucrose–phosphate–glutamate, pH 7.2 (SPG), and slowly frozen to −70 °C. Blind passage was done for *C. trachomatis* NAAT positive specimens—the cells collected previously in SPG were re-inoculated into McCoy cells (wells with glass coverslips and wells as such) with the procedure described above. The growth of *C. trachomatis* was detected by direct immunofluorescence staining with a Pathfinder *Chlamydia* Culture Confirmation System (Bio-Rad, Hercules, CA, USA). The *C. trachomatis* culture positive isolates were genotyped using the method described earlier [20], with modifications [21].

Five low-passage-number (passage ≤5) clinical isolates representing the most common genotypes of E and F (E127, E129, E142, F175, and F213) were included in this analysis [21]. The characteristics of the patients whose isolates were studied are presented in Table 1. These clinical isolates as well as reference strains of type E (Bour, VR-348B) and type F (IC-Cal-3, VR-346) (from American Type Culture Collection, ATCC, Manassas, VA, USA) were inoculated onto HeLa229 cells (ATCC CCL-2.1) in 24-well plates at a multiplicity of infection (MOI) of 1.0. The plates were centrifuged at 3000× *g* at 30 °C for one hour and incubated in 5% CO_2_ at 35 °C for one hour. Minimum Essential Medium Eagle Alpha Modification (α-MEM; Sigma-Aldrich, Darmstadt, Germany) medium supplemented with 10% fetal calf serum, 2 mM glutamine (GlutaMAX, Gibco, Thermo Fisher Scientific, Waltham, MA, USA), 0.5% glucose, 20 μg/mL gentamicin, 50 U/mL nystatin, and 0.5 μg/mL cycloheximide was added, and the plates were incubated in 5% CO_2_ at 35 °C. After incubation for 2, 6, 12, 24, 36, and 48 hours, the cells were collected in ice-cold phosphate-buffered saline. The experiment included two technical replicates of the infected cells, and it was repeated once.

### 2.3. Nucleic Acid Extraction and cDNA Synthesis

The DNA was extracted from *C. trachomatis* infected McCoy cells (passage 3 to ≤5) with a DNeasy blood and tissue kit (Qiagen, Hilden, Germany) for *C. trachomatis* genotyping. The DNA was extracted from infected HeLa229 cells with a PureLink Genomic DNA Kit (Thermo Fisher Scientific) for quantitating *C. trachomatis* genome equivalents (GEs). The RNA was extracted from an aliquot of infected HeLa229 cells stored in a RNAlater Solution (Thermo Fisher Scientific) with RNAqueous-4PCR Kit (Thermo Fisher Scientific) and treated with two units of DNase using the TURBO-DNA-free Kit (Thermo Fisher Scientific) to remove trace DNA contamination. In addition, RNA was extracted from 44 *C. trachomatis* NAAT positive and 8 NAAT negative cervical swabs in the Aptima Swab Transport Medium (Hologic) with a RNAqueous-4PCR Kit (Thermo Fisher Scientific).

cDNA was synthesized with a Maxima First Strand cDNA Synthesis Kit for RT-qPCR (Thermo Fisher Scientific) using the RNA extracted from HeLa229 cells and cervical samples. A reverse transcriptase negative (RT-) control was included for every sample. No-template control was used to assess for reagent contamination.

### 2.4. Real-Time PCR

*C. trachomatis ompA* PCR was performed with a method designed previously [22]. The primers and probes for the *C. trachomatis cpaf*, *tarp*, and *tox* PCRs were designed with Primer Express software version 3.0 (Applied Biosystems, Thermo Fisher Scientific), based on the complete genome sequences of *C. trachomatis* (NCBI Reference Sequence Database). The primers and probes were tested for specificity by the basic local alignment search tool (BLAST). The primers and probes were purchased from Applied Biosystems, Metabion and Oligomer. The primer and probe sequences and amplicon lengths are presented in Table 2.

Real-time PCRs were performed in a 25 μL volume containing 12.5 μL Maxima Probe qPCR Master Mix (Thermo Fisher Scientific). The PCR for *cpaf* contained 600 nM primers and 200 nM probes, for *tarp* the PCR contained 300 nM primers and 200 nM probes, and for *tox* the PCR contained 600 nM primers and 100 nM probes. The PCR analyses were performed on an ABI 7500 instrument and Sequence Detection Software version 1.3.1 (Applied Biosystems, Thermo Fisher Scientific). The thermal cycling conditions were two minutes at 50 °C, ten minutes at 95 °C, 40 cycles of 15 seconds at 95 °C, and one minute at 60 °C. The template volume was 2 μL of DNA and cDNA, and each sample was amplified in duplicate.

To evaluate the performance of the *C. trachomatis cpaf*, *tarp*, and *tox* PCRs, DNA from *C. trachomatis* reference strains types A-L2 (ATCC numbers A:VR-571B, B:VR-573, C:VR-572, D:VR-885, E:VR-348B, F:VR-346, G:VR-878, H:VR-879, I:VR-880, J:VR-886, K:VR-887, and L2:VR-903) propagated in McCoy cells; *C. pneumoniae* isolate Kajaani-6 (from Pekka Saikku, University of Oulu, Finland) propagated in HL cells; eukaryotic cell lines A549, HL, and McCoy; and ten cervical swabs cultivated on agar plates (representing cervical bacterial flora) were tested. DNA was extracted from the bacteria on agar plates, as previously discussed [23]. The DNA was extracted from the other samples with either a MagNA Pure Compact instrument (Roche Diagnostics GmbH, Mannheim, Germany) using MagNA Pure Compact Nucleic Acid Isolation Kit I (Roche Diagnostics GmbH), or with a DNeasy blood and tissue kit (Qiagen).

To quantify the *C. trachomatis* GEs during HeLa229 cell infection, qPCR was applied to the extracted DNA and an external plasmid standard. A pIDTSMART-AMP plasmid containing the *ompA* PCR target sequence of *C. trachomatis* reference strain type E (Bour) was purchased from Integrated DNA Technologies (Coralville, IA, USA). Serially ten-fold diluted plasmid DNA (10^3^–10^8^ copies/reaction) was amplified by the *ompA* PCR. The standard curve was generated by plotting the *C*t values against the known initial plasmid copy numbers. The generation (doubling) time (i.e., the time interval required for the bacteria to divide) was calculated according to a formula from Todar´s Online Textbook of Bacteriology [24].

Real-time PCR was applied to study the *ompA*, *cpaf*, *tarp*, and *tox* expression. As an amplification control and a relative standard, serially ten-fold diluted *C. trachomatis* reference strain type E (Bour) DNA (10^−1^–10^−6^) was used. At each time after infection, the raw *ompA*, *cpaf*, *tarp,* and *tox* PCR amplification data was normalized against the number of *C. trachomatis* GEs in each sample [25]. To detect chlamydial genomic DNA contamination and additional RT- control was amplified with every sample. No-template control was included in each run, to assess for reagent contamination.

## 3. Results

### 3.1. Clinical C. trachomatis Isolates

Of the swabs from the 50 *C. trachomatis* NAAT positive females, we could culture 40 (80%) *C. trachomatis* isolates. Genotypes E (40%), F (20%), and G (13%) were the three most common. The genotype distribution was rather similar to the one described by us earlier [21]. The genotyping results have been confirmed by the whole genome sequencing of the isolates (the sequencing data have been deposited in the European Nucleotide Archive, ENA, http://www.ebi.ac.uk/ena) [26].

### 3.2. Growth Kinetics of C. trachomatis Reference Strains and Clinical Isolates

**Figure 1:**
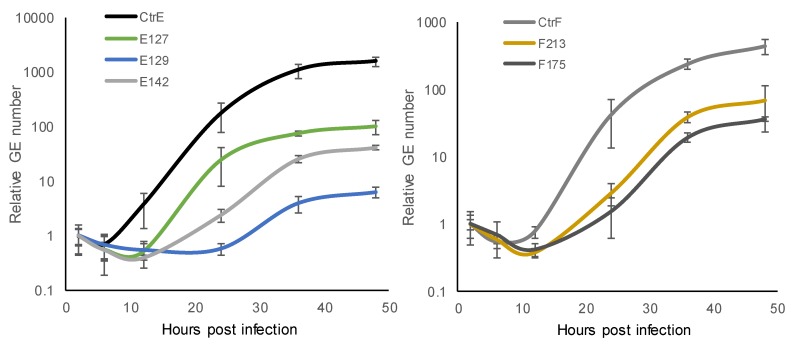
Growth kinetics of *C. trachomatis* reference strain type E (CtrE) and clinical isolates type E (E127, E129, and E142), and reference strain type F (CtrF) and clinical isolates type F (F213 and F175) at 2, 6, 12, 24, 36, and 48 hours post infection (hpi) in HeLa229 cells determined with *ompA* PCR. The stages of the chlamydial developmental cycle include early- (2–6 hpi), mid- (6–24 hpi), and late-stage (24–48 hpi). The results are shown as relative genome equivalents (GEs; the number of GEs at 2 hpi was set as one). The results are based on two experiments, both including two technical replicates.

At each time-point, the number of *C. trachomatis* GEs in the HeLa229 cultures was determined by *ompA* qPCR. The results are based on two technical replicates, and the experiment was repeated once with similar results. One-step growth curves showed that the exponential period of growth (exponential change in GEs) in the *C. trachomatis* reference strain type E and F took place at 12–36 hpi (Figure 1). In the clinical isolates, the exponential period of growth took place at 12–24 hpi in E127, at 12–36 hpi in E142, F213 and F175, and at 24–36 hpi in E129.

The generation time (time interval required for the division of bacteria) was calculated. A comparison of the number of GEs at the beginning and at the end of the exponential growth phase of *C. trachomatis* reference strain type E suggested a doubling time of 2.8 h, for the reference strain type F 2.9 h, and for the clinical isolate E127 2.2 h, whereas for the other clinical isolates, the doubling time was longer (E129 4.3 h, E142 4.0 h, F213 3.6 h, and F175 4.3 h). All of the clinical isolates, except one (E127), exhibited a slower growth rate than the reference strains.

During the developmental cycle after the lag phase (6–12 hpi), the number of *C. trachomatis* GEs increased considerably when the reference strains were evaluated (2200-fold in *C. trachomatis* reference strain type E and 790-fold in reference strain type F). The overall fold-increase in the GEs of the clinical isolates was smaller, with an 11- to 190-fold increase in types E, and a 90- to 180-fold increase in types F. The reference strains propagated more efficiently progeny than the clinical isolates.

### 3.3. Performance of C. trachomatis cpaf, tarp, and tox Real-Time PCRs

When *C. trachomatis* reference strain type E (Bour) DNA was amplified, the limit of detection for *cpaf*, *tarp* and *tox* PCRs corresponded to 1.6 inclusion forming units per reaction. Standard curve construction showed a linear relation between log values of DNA and PCR threshold cycles over five orders of magnitude. The efficiency of amplification was 99% (slope −3.358) for *cpaf*, 97% (slope −3.408) for *tarp* and 83% (slope −3.811) for *tox* PCR. To test for the specificity of the assays, DNA from *C. trachomatis* reference strains types A-L2, C. pneumoniae K6, cell lines A549, HL and McCoy, and ten cervical samples grown on agar plates were analyzed. The *tarp* and *cpaf* PCRs detected DNA from all *C. trachomatis* types while the rest of the samples tested negative. As expected, DNA from *C. trachomatis* types A, C and D-K tested positive by *tox* PCR, and the rest of the samples remained negative. In *C. trachomatis* type B, the entire cytotoxin gene region is deleted from the genome, and in types L1-3, there is an extensive deletion in the cytotoxin gene [27].

### 3.4. Expression of C. trachomatis ompA, cpaf, tarp, and tox Genes In Vitro

**Figure 2:**
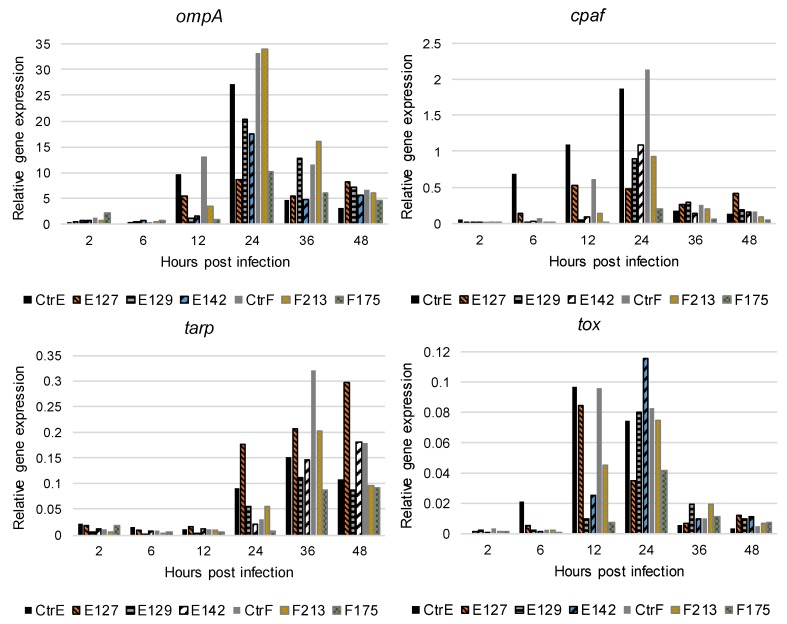
The expression of the *ompA*, *cpaf*, *tarp*, and *tox* genes of *C. trachomatis* reference strain type E (CtrE), reference strain type F (CtrF), and five clinical isolates types of E and F (E127, E129, E142, F213, and F175) at 2, 6, 12, 24, 36, and 48 hours post infection in HeLa229 cells. The results are based on two technical replicates and the experiment was repeated once with similar results. At each time after infection, raw *ompA*, *cpaf*, *tarp*, and *tox* PCR amplification data were normalized against the number of *C. trachomatis* genome equivalents (GEs) in each sample [25].

The expression of the selected *C. trachomatis* genes was analyzed in infected HeLa229 cells at 2, 6, 12, 24, 36, and 48 hpi. The results are based on two technical replicates and the experiment was repeated once, with similar results. *C. trachomatis ompA* was expressed between 12 and 48 hpi, and the peak of the expression was observed at 24 hpi in the reference strains and the clinical isolates (Figure 2). Among the reference strains and the clinical isolate E127, the *ompA* expression was high already at 12 hpi, compared with the other clinical isolates.

The expression of *cpaf* increased and peaked at 12 hpi in the clinical isolate E127, and at 24 hpi in the reference strains and the other clinical isolates. In addition, the *cpaf* expression was high at 6, 12, and 24 hpi in the reference strain type E and at 24 hpi in the reference strain type F, compared with the clinical isolates. The expression of *tarp* was observed between 24 and 48 hpi. The reference strains and the clinical isolates had a rather similar *tarp* expression profile (Figure 2). The expression of *tox* was observed between 12 and 48 hpi, and the peak of the expression was at 12 hpi in the reference strains and the clinical isolate E127, and at 24 hpi in the other clinical isolates.

The DNA and cDNA from the uninfected HeLa229 cell cultures were negative for all of the PCRs. The no-template control remained negative for all of the PCRs. Most of the RT- controls remained negative by PCR. However, some RT- controls from timepoints 36 and 48 hpi gave weak signals late in the PCR (Ct > 37).

### 3.5. Expression of C. trachomatis ompA, cpaf, tarp, and tox Genes in the Cervical Swabs

Next, we attempted to analyze the transcriptional expression of the selected *C. trachomatis* genes directly in the cervical swab specimens. Small amounts of mRNA of one or several of the genes were detected in nine out of forty-four *C. trachomatis* NAAT positive samples—*ompA* mRNA in five, *cpaf* mRNA in five, *tarp* mRNA in three, and *tox* mRNA in three samples. In the eight *C. trachomatis* NAAT negative samples, no mRNA was detected.

## 4. Discussion

The long-term propagation of *C. trachomatis* in the cell culture can alter the regulation of the bacterial gene expression and lead to a faster growth rate [16,17,18]. For this study, low-passage-number *C. trachomatis* clinical isolates of two different genotypes were obtained from females attending an STI clinic. The growth characteristics and expression patterns of four genes among the five clinical isolates and two reference strains were analysed by PCR-based analyses. In agreement with earlier findings, the reference strains of type E and F exhibited a faster growth rate than four out of the five clinical low-passage-number isolates studied. Indeed, a doubling (generation) time of approximately two to three hours was estimated for the reference strains and one clinical isolate, whereas the other clinical isolates had a doubling time of approximately four hours. Likewise, a generation or doubling time of two to three hours has been estimated in urogenital *C. trachomatis* strains by others [25,28]. In addition, the reference strains propagated more efficiently than the clinical isolates in the cell culture conditions. These observed changes reflect the adaptation of multiple times passaged *C. trachomatis* reference strains to in vitro conditions [18,19]. This suggests that the use of low-passage-number isolates in experimental approaches might better mimic the situation during infection. However, to our surprise, one of the low-passage-number isolates, E127, exhibited characteristics similar to the reference strains. The isolate was cultured from a cervical swab of an asymptomatic, seronegative female, whose partner had tested *C. trachomatis* positive (Table 1). The clinical data available suggests that she could be experiencing her first *C. trachomatis* infection, but the ultimate factors explaining the growth properties of the bacteria remain undefined. The other isolates studied were from *C. trachomatis* seropositive females suffering from or possibly having repeated *C. trachomatis* infection.

For the first time, the in vitro expression of the *C. trachomatis cpaf*, *tarp*, and *tox* genes was studied at different stages of the chlamydial developmental cycle using low-passage-number clinical isolates of types E and F. We observed dissimilarities in the *C. trachomatis* gene expression profile between the low-passage-number clinical isolates and the reference strains. The expression of *cpaf* was high at the mid-phase of the developmental cycle in the reference strains, compared with the clinical isolates. The relevance of this remains unknown, as the precise target molecules of CPAF and the role of this protease during chlamydial infection are under reevaluation [12]. The peak of expression for *tox* (at 12 hpi) was also observed earlier in the reference strains than for most clinical isolates (24 hpi). The toxin has a role early during developmental cycle [13], so this might also associate with the faster growth of these strains. Only the expression profile of *tarp* was rather similar among the clinical isolates and the reference strains. The expression of *tarp* was observed later than that of the other genes. Indeed, in earlier studies, the type L2 *tarp* expression was categorized as late (24–36 hpi) [6] or type D *tarp* expressed at 8–40 hpi [7].

The expression of *ompA* (starting at 12 hpi) took place earlier in the reference strains and one clinical isolate than in the other clinical isolates. This might reflect the role of MOMP as a major structural component of the cell membrane in dividing RBs. The observed variation in the transcriptional expression among the clinical isolates is probably due to the intrinsic properties of the single isolates because the growth conditions were identical. Infected HeLa229 cell cultures were used in these experiments, as they represent the most established *C. trachomatis* infection models and have extensively been used previously [19,29]. Moreover, the *ompA* expression data obtained with the reference strains in this model were in line with the earlier data [7,19,30], suggesting that the infection model used by us is comparable to those used in the previous studies. In some previous studies, the data were achieved with microarray or RNA sequencing techniques. However, it has been demonstrated that the RT-PCR data correlates well with the data obtained by both techniques [6,7,31].

The mRNA of the *C. trachomatis ompA*, *tox*, *tarp*, and *cpaf* genes was infrequently detected in the cervical swabs, although the corresponding low-passage-number isolates readily expressed these genes in vitro. This suggests that the absence of a signal in the cervical swabs can be due to the small amount of chlamydial mRNA in the samples, and not to a variation in gene expression during in vivo infection. This is further supported by serology, in which the responses to the proteins encoded by genes *ompA*, *cpaf*, *tarp*, and *tox* were observed in the sera from *C. trachomatis* seropositive patients (our unpublished observation). The few studies on the in vivo gene expression of *C. trachomatis* analyzed that the cytobrush cervical samples [32] or synovial biopsies [33] probably contain more *C. trachomatis* cells than cervical swabs. Moreover, as *C. trachomatis* could be cultured, the swab specimens contained chlamydial EBs, but the presence of RBs (metabolically active forms) remains unknown.

The strengths of our approach include the use of characterized, low-passage-number *C. trachomatis* clinical isolates, and an established cell culture model of infection. Also, we used the relative quantitation of cDNA, which allows for the comparison of the gene expression between different timepoints. We chose to use chlamydial genomic DNA in the normalization of the gene expression data. Although the number of genomes per bacteria might vary slightly during replication, this approach has proven appropriate [25,34,35]. One weakness is that the determination of the mRNA and genomic DNA are experimentally independent, which could create a bias in the gene expression patterns. Also, the mRNA decay rate was not analyzed.

Studies on the *C. trachomatis* gene expression and growth kinetics during in vitro and in vivo infection promote an understanding of the disease pathogenesis, as well as finding novel therapeutic options. Transcriptomics remain an essential tool in such studies. In the future, the challenges in the in vivo gene expression studies of *C. trachomatis* may be overcome with the development of more sensitive transcriptome analysis methods such as single-cell RNA sequencing [36]. Here, the *C. trachomatis* low-passage-number clinical isolates acted differently compared to the reference strains. This calls for the careful evaluation of the strains used in chlamydial research. In general, the use of characterized *C. trachomatis* low-passage-number isolates is encouraged, as it could better reflect the situation in vivo during culture-positive infection than the use of the readily available reference strains.

## Figures and Tables

**Table 1:** Characteristics of the five patients whose *C. trachomatis* isolates were included in the in vitro gene expression assays.

Isolate	Age	Sex	Specimen Type	*ompA* Genotype	Ctr Serology IgG/IgM/IgA ^1^	Clinical Features	Other STIs (Gonorrhea, HIV, or Syphilis)
E127	27	F	Cervical swab	E	<32/<20/<20	Asymptomatic, partner infected, probable first Ctr infection ^2^	none
E129	20	F	Cervical swab	E	128/<20/<20	Asymptomatic, partner infected, probable repeated Ctr infection ^3^	none
E142	20	F	Cervical swab	E	32/<20/<20	Pain in the lower abdomen, vaginal discharge, BV, repeated Ctr infection ^4^	none
F175	24	F	Cervical swab	F	32/<20/<20	Asymptomatic, BV, partner infected, repeated Ctr infection ^4^	none
F213	21	F	Cervical swab	F	128/<20/<20	Asymptomatic, probable repeated Ctr infection ^3^	none

BV—bacterial vaginosis; Ctr—*C. trachomatis*; STI—sexually transmitted infection; HIV—human immunodeficiency virus; Sex F—female; ^1^ MIF (micro-immunofluorescence) serology; ^2^ Negative *C. trachomatis* NAATs in history, and immunoglobulin G (IgG) antibody negative; ^3^ Negative *C. trachomatis* nucleic acid amplification tests (NAATs) in history, but IgG antibody positive; ^4^ Positive *C. trachomatis* NAATs in history.

**Table 2:** Sequences of the *cpaf*, *tarp*, *tox*, and *ompA* primers and probes used in the real-time PCR analysis.

Ctr Target Gene	Amplicon Length	Reference	Primer/Probe (5′–3′)	
*cpaf*	86 bp	This study	Forward	TAGGATGGGATCTTGTTCAAAGCT
			Reverse	CTGCTGGCAAAAACTTGTTGAT
			Probe	6-FAM-CTGCACAGCAGAAGCTTCGTACACAAGAA-BHQ-1
*tarp*	108 bp	This study	Forward	CCTCTTCTGGAGATGATTCAGGAA
			Reverse	TACGCACGGCAGAAAGGATA
			Probe	6-FAM-CCTCTGTCGGAAATGACGGACCTGCT-BHQ-1
*tox*	106 bp	This study	Forward	GATTCTTTAATTTCTGCTTGCTGAAA
			Reverse	TGTTCGATCTCCTCAGTAGGAAGTTT
			Probe	6-FAM-CTCGGCAATATCAATGACGAAACGCGT-BHQ-1
*ompA*	219 bp	[22]	Forward	GACTTTGTTTTCGACCGTGTT
			Reverse	ACARAATACATCAAARCGATCCCA
			Probe	VIC-ATGTTTACVAAYGCYGCTT-MGB-NFQ

Ctr—*C. trachomatis*.
